# Outcomes of Minimally Invasive Aortic Valve Replacement in Obese Patients: A Propensity-Matched Study

**DOI:** 10.21470/1678-9741-2023-0159

**Published:** 2024-02-06

**Authors:** Federico Cammertoni, Piergiorgio Bruno, Natalia Pavone, Marialisa Nesta, Giovanni Alfonso Chiariello, Maria Grandinetti, Serena D’Avino, Valerio Sanesi, Denise D’Errico, Massimo Massetti

**Affiliations:** 1 Department of Cardiovascular Sciences, Cardiac Surgery Unit, Fondazione Policlinico Universitario Agostino Gemelli IRCCS, Rome, Italy; 2 Catholic University of the Sacred Heart, Rome, Italy; 3 Department of Cardiovascular Sciences, Perfusion Unit, Fondazione Policlinico Universitario Agostino Gemelli IRCCS, Rome, Italy

**Keywords:** Aortic Valve, Obesity, Minimally Invasive Surgical Procedures, Artificial Respiration, Length of Stay, Airway Extubation, Postoperative Complications

## Abstract

**Introduction:**

Obese patients are at risk of complications after cardiac surgery. The aim of this study is to investigate safety and efficacy of a minimally invasive approach via upper sternotomy in this setting.

**Methods:**

We retrospectively reviewed 203 obese patients who underwent isolated, elective aortic valve replacement between January 2014 and January 2023 - 106 with minimally invasive aortic valve replacement (MIAVR) and 97 with conventional aortic valve replacement (CAVR). To account for baseline differences, a propensity-matching analysis was performed obtaining two balanced groups of 91 patients each.

**Results:**

The 30-day mortality rate was comparable between groups (1.1% MIAVR vs. 0% CAVR, P=0.99). MIAVR patients had faster extubation than CAVR patients (6 ± 2 vs. 9 ± 2 hours, P<0.01). Continuous positive airway pressure therapy was less common in the MIAVR than in the CAVR group (3.3% vs. 13.2%, P=0.03). Other postoperative complications did not differ significantly. Intensive care unit stay (1.8 ± 1.2 vs. 3.2 ± 1.4 days, P<0.01), but not hospital stay (6.7 ± 2.1 vs. 7.2 ± 1.9 days, P=0.09), was shorter for MIAVR than for CAVR patients. Follow-up survival was comparable (logrank P-value = 0.58).

**Conclusion:**

MIAVR via upper sternotomy has been shown to be a safe and effective option for obese patients. Respiratory outcome was promising with shorter mechanical ventilation time and reduced need for post-extubation support. The length of stay in the intensive care unit was reduced. These advantages might be important for the obese patient to whom minimally invasive surgery should not be denied.

## INTRODUCTION

**Table:** 

Abbreviations, Acronyms & Symbols				
ACC	= Aortic cross-clamping		ICU	= Intensive care unit
AF	= Atrial fibrillation		IQR	= Interquartile range
BMI	= Body mass index		LVEF	= Left ventricular ejection fraction
CAVR	= Conventional aortic valve replacement		MIAVR	= Minimally invasive aortic valve replacement
COPD	= Chronic obstructive pulmonary disease		NYHA	= New York Heart Association
CPAP	= Continuous positive airway pressure		PMK	= Permanent pacemaker
CPB	= Cardiopulmonary bypass		PVD	= Peripheral vascular disease
eGFR	= Estimated glomerular filtration rate		STS	= Society of Thoracic Surgeons

Obesity (body mass index [BMI] ≥ 30 kg/m^2^) is an emerging public health problem in the Western world^[1]^. Several authors have tried to clarify how obesity affects the outcome of patients undergoing cardiac surgery, but the results have been mixed. Indeed, some found an unexpected protective effect (obesity paradox) and associated obesity with a lower postoperative risk^[2]^. Others have refuted these findings, concluding that being overweight increases complications after cardiac surgery^[3]^. Thus, whether the “obesity paradox” exists only in clinical studies or also has an effect in the “real world” has not yet been conclusively established^[4]^.

Beyond this, agreement remains on the fact that, both during surgery and in the immediate postoperative period, obese patients are challenging^[5,6]^.

Although still under debate, substantial evidence suggests that minimally invasive surgery is associated with certain advantages: faster recovery of respiratory function, less postoperative pain, reduced bleeding and less need for transfusions, shorter intensive care unit (ICU) and hospital stay, and faster functional recovery^[7]^. All these benefits would be desirable for obese patients^[8,9]^. However, few and largely dissimilar studies have attempted to answer the question of whether obese patients would benefit from a minimally invasive approach compared to a conventional one. This lack of evidence and the fear of suboptimal surgical exposure resulting in prolonged operating times could jeopardise the use of minimally invasive surgery in these patients^[9,11]^, as evident from a survey by Misfeld et al.^[12]^.

The objective of this propensity-matched study is to investigate safety and efficacy of a minimally invasive partial upper sternotomy approach and to test whether it confers a clinical advantage over full sternotomy in obese patients who are candidates for isolated aortic valve surgery.

## METHODS

This study was approved by the institutional research ethics committee (protocol: 0016081/22). The need for informed patient consent was waived because of the retrospective study design. This study has been conducted in accordance with the principles set forth in the Helsinki Declaration.

### Patients

From January 2014 to January 2023, 723 patients underwent elective, isolated aortic valve replacement at our Centre. In order to reduce possible confounding factors, we excluded patients with active infective endocarditis and those who needed redo surgery. Finally, we only considered obese patients, *i.e.*, with BMI ≥ 30 kg/m^2^. We thus obtained 203 patients: 106 (52.2%) underwent minimally invasive surgery through of a partial upper sternotomy (minimally invasive aortic valve replacement [MIAVR] group) and 97 (47.8%) underwent conventional full sternotomy surgery (conventional aortic valve replacement [CAVR] group). Preoperative, intraoperative, and postoperative data were retrospectively retrieved from the local Heart Valve Database. Conversions to full sternotomy were assigned to the MIAVR group for an intention-to-treat analysis. In February 2023, patients or their referral physicians were contacted by telephone for clinical follow-up.

The Heart Team determined surgical indication whereas the decision between CAVR and MIAVR was left to the surgeon. All procedures were performed by four surgeons, equally skilled in conventional and minimally invasive surgery. Severe chest deformities, ascending aorta calcifications, and previous chest irradiation were contraindications for minimally invasive surgery.

### Outcomes

The primary outcome was 30-day mortality. Secondary outcomes were: duration of mechanical ventilation, need for reintubation, need for continuous positive airway pressure (CPAP) therapy, need for inotropic support, postoperative stroke, peripheral vascular complications, need for red blood cells transfusion, superficial wound complications, deep sternal wound complications, postoperative bleeding requiring surgical revision, pacemaker implantation, postoperative atrial fibrillation, need for dialysis, and ICU and hospital length of stay. The overall duration of surgery, extracorporeal circulation, and aortic cross-clamping were compared between the two groups. Finally, mortality at follow-up was compared.

### Surgical Technique for Minimally Invasive Surgery

MIAVR patients underwent a 4-5 cm skin incision and partial upper V-shaped sternotomy, from the jugular notch to the fifth intercostal space. Arterial cannulation was in the ascending aorta. Venous cannulation was systematically performed percutaneously via the right common femoral vein. Left heart venting was carried out either percutaneously using a dedicated pulmonary vent (EndoVent®, Edwards Lifesciences, Irvine, California, United States of America) or in the traditional fashion through the right upper pulmonary vein.

In case of more than mild aortic regurgitation, retrograde cardioplegia delivery was achieved using a specific catheter (ProPlege®, Edwards Lifesciences, Irvine, California, United States of America). Both devices were placed before surgical incision by cardiac anaesthesiologists. All percutaneous cannulations were ultrasound guided. In the hybrid operating theatre, fluoroscopic imaging was also used. Carbon dioxide continuously flooded the operative field to decrease the risk of air embolism.

More details on surgical technique, extracorporeal circulation setup, and anaesthesia management have been previously described^[13,14]^.

### Statistical Analysis

Continuous variables are shown as mean ± standard deviation if normally distributed and as median (interquartile range [IQR]) otherwise. Percentages are used to describe categorical variables. Kolmogorov-Smirnov test was used to check the normality/skewness of continuous variables before further analysis. Groups were compared using the Fisher’s exact test or χ^2^ test for categorical variables, as appropriate. Continuous variables were compared using independent samples *t*-test or Mann-Whitney U test, as appropriate. All tests were two-sided and a type I error significance level of 0.05 was considered. Missing data were replaced by the mean if their percentage was < 5% for the variable in question. If the number of missing data was > 5%, a listwise deletion method was adopted. To reduce the effect of selection bias, we resorted to a propensity-matching analysis. A propensity score, indicating the predicted probability of receiving MIAVR, was calculated with multiple logistic regression using all variables listed in Table 1. Then, we matched MIAVR to CAVR patients using a 1:1 nearest-neighbour matching with a 0.1 caliper and no replacement. Survival was analysed using the Kaplan-Meier method, and the groups were compared using the logrank test. Data analysis was performed with SPSS Statistics version 19.0 (IBM Corporation, Armonk, NY).

**Table 1:** Baseline characteristics of the unmatched and propensity-matched groups.

	Before matching (n = 203)			Propensity-matched groups (n = 182)		
	MIAVR group (n=106)	CAVR group (n=97)	*P*-value	MIAVR group (n=91)	CAVR group (n=91)	*P*-value
Age, years	68.9 ± 15.1	70.8 ± 13.2	0.34	68.6 ± 14.5	69.2 ± 13.1	0.77
Age ^3^ 80 years	11 (10.4%)	13 (13.4%)	0.52	9 (9.9%)	10 (11.0%)	0.99
Male gender	63 (59.4%)	60 (61.9%)	0.77	55 (60.4%)	54 (59.3%)	0.99
BMI, kg/m^2^	33.1 ± 1.2	32.8 ± 1.1	0.07	32.9 ± 1.3	32.6 ± 1.2	0.11
Haemoglobin, g/dL	13.1 ± 1.6	13.7 ± 1.5	0.01	13.2 ± 1.4	13.5 ± 1.3	0.14
NYHA class ^3^ III	22 (20.8%)	33 (34.0%)	0.04	21 (23.1%)	27 (29.7%)	0.40
Syncope	6 (5.7%)	12 (12.4%)	0.14	5 (5.5%)	8 (8.8%)	0.57
Active smoker	29 (27.4%)	26 (26.8%)	0.99	26 (28.6%)	24 (26.4%)	0.87
Hypertension	81 (76.4%)	79 (81.4%)	0.40	76 (83.5%)	75 (82.4%)	0.99
Diabetes mellitus	21 (19.8%)	25 (25.8%)	0.32	18 (19.8%)	22 (24.2%)	0.59
Dyslipidemia	48 (45.3%)	47 (48.5%)	0.67	42 (46.2%)	44 (48.4%)	0.88
COPD	4 (3.8%)	12 (12.4%)	0.03	4 (4.4%)	7 (7.7%)	0.53
PVD	6 (5.7%)	12 (12.4%)	0.14	5 (5.5%)	7 (7.7%)	0.77
Previous stroke	3 (3.3%)	4 (4.1%)	0.71	2 (2.2%)	4 (4.4%)	0.68
eGFR^*^ < 50 ml/h	3 (2.8%)	10 (10.3%)	0.04	2 (2.2%)	5 (5.2%)	0.44
Atrial fibrillation	13 (12.3%)	15 (15.5%)	0.55	9 (9.9%)	10 (11.0%)	0.99
LVEF, %	62.1 ± 6.8	59.3 ± 5.8	< 0.01	61.3 ± 6.2	60.1 ± 5.5	0.17
STS score	1.61 ± 0.58	2.35 ± 0.61	< 0.01	1.54 ± 0.53	1.69 ± 0.59	0.07

* Cockcroft and Gault formula

Data were presented as n (%) or mean ± standard deviation

BMI=body mass index; CAVR=conventional aortic valve replacement; COPD=chronic obstructive pulmonary disease; eGFR=estimated glomerular filtration rate; LVEF=left ventricular ejection fraction; MIAVR=minimally invasive aortic valve replacement; NYHA=New York Heart Association; PVD=peripheral vascular disease; STS=Society of Thoracic Surgeons

## RESULTS

### Baseline Characteristics

Table 1 shows the demographic and clinical characteristics of the two groups both before and after matching. At baseline, CAVR patients had worse symptoms than MIAVR patients (New York Heart Association class ≥ III: 34.0% *vs.* 20.8%, *P*=0.04), higher incidence of severe chronic kidney disease (10.3% *vs.* 2.8%, *P*=0.04) and chronic obstructive pulmonary disease (12.4% *vs.* 3.8%, *P*=0.03), lower left ventricular ejection fraction (59.3 ± 5.8 *vs.* 62.1 ± 6.8, *P*<0.01), and higher surgical risk as estimated with the Society of Thoracic Surgeons score (2.35 ± 0.61 *vs.* 1.61 ± 0.58, *P*<0.01).

After propensity-matching, we obtained two homogeneous groups of 91 patients each, with well-balanced baseline characteristics.

As described in Table 2, degenerative heart valve disease was the most common cause of aortic valve defect, with no differences between the groups (52.7% MIAVR *vs.* 55.0% CAVR, *P*=0.88). Mainly, patients had isolated aortic valve stenosis (52.7% in the MIAVR group *vs.* 62.6% in the CAVR group, *P*=0.23); combined defects (31.9% MIAVR *vs.* 19.8% CAVR, *P*=0.09) and isolated aortic valve regurgitation (15.4% MIAVR *vs.* 17.6% CAVR, *P*=0.84) were less common.

**Table 2:** Propensity-matched groups comparison of type of valve disfunction and etiology.

	Propensity-matched groups (n = 182)		
	MIAVR group (n=91)	CAVR group (n=91)	*P*-value
Valve disfunction			
Isolated aortic stenosis	48 (52.7%)	57 (62.6%)	0.23
Isolated aortic regurgitation	14 (15.4%)	16 (17.6%)	0.84
Mixed stenosis and regurgitation^*^	29 (31.9%)	18 (19.8%)	0.09
Etiology			
Degenerative	48 (52.7%)	50 (55.0%)	0.88
Bicuspid	31 (34.1%)	24 (26.4%)	0.33
Rheumatic	10 (11.0%)	9 (9.9%)	0.99
Previous endocarditis	0 (0%)	2 (2.2%)	0.50
Cusp prolapse	0 (0%)	1 (1.1%)	0.99
Combination	2 (2.2%)	5 (5.4%)	0.44

Data were presented as n (%)

^*^Severe aortic valve stenosis or regurgitation associated with at least moderate aortic valve regurgitation or stenosis, respectively

CAVR=conventional aortic valve replacement; MIAVR=minimally invasive aortic valve replacement

### Surgical Outcomes

Percutaneous femoral vein, pulmonary artery, and coronary sinus cannulation were used in 96.7%, 78.0%, and 18.7% of MIAVR patients, respectively (Table 3). Conversion to full sternotomy was required in three MIAVR patients (3.3%) following ineffective attempts to cannulate the femoral vein. Minimally invasive surgery required both significantly longer extracorporeal circulation time (109.6 ± 17.5 *vs.* 98.6 ± 16.2 minutes, *P*<0.01) and total surgery time (246.6 ± 32.1 vs. 221.4 ± 33.4 minutes, *P*<0.01) than the conventional approach. However, the duration of aortic cross-clamping did not differ significantly (69.6 ± 14.1 *vs.* 72.1 ± 15.6 minutes for MIAVR and CAVR groups, respectively, *P*=0.26).

**Table 3:** Propensity-matched groups comparison of surgical outcomes.

	Propensity-matched groups (n = 182)		
	MIAVR group (n=91)	CAVR group (n=91)	*P*-value
CPB time, min.	109.6 ± 17.5	98.6 ± 16.2	< 0.01
ACC time, min.	69.6 ± 14.1	72.1 ± 15.6	0.26
Surgery time min.	246.6 ± 32.1	221.4 ± 33.4	< 0.01
Conversion to full sternotomy	3 (3.3%)	-	-
Percutaneous femoral vein	88 (96.7%)	-	-
EndoVent®^*^	71 (78.0%)	-	-
ProPlege®^*^	17 (18.7%)	-	-

Data were presented as n (%) or mean ± standard deviation

^*^EndoVent® and ProPlege® (Edwards Lifesciences, Irvine, California, United States of America)

ACC=aortic cross-clamping; CAVR=conventional aortic valve replacement; CPB=cardiopulmonary bypass; MIAVR=minimally invasive aortic valve replacement

### Clinical Outcomes

The 30-day mortality rate did not differ between the two groups (1.1% MIAVR *vs.* 0% CAVR, *P*=0.99) (Table 4). One patient in the MIAVR group died of stroke 20 days after surgery during postoperative rehabilitation. Patients in the MIAVR group had faster extubation (6 ± 2 *vs.* 9 ± 2 hours, *P*<0.01) and required CPAP therapy less (3.3% *vs.* 13.2%, *P*=0.03). A trend towards higher rate of reintubation (5.5% CAVR *vs.* 1.1% MIAVR, *P*=0.21) and red blood cells transfusion (24.2% *vs.* 16.5%, *P*=0.27) was also observed. Other major postoperative complications did not differ significantly between the two groups. ICU stay was shorter for MIAVR patients (1.8 ± 1.2 *vs.* 3.2 ± 1.4 days, *P*<0.01). Finally, we found no differences in the duration of hospital stay (6.7 ± 2.1 *vs.* 7.2 ± 1.9 days, *P*=0.09).

**Table 4:** Postoperative outcomes of the propensity-matched groups.

	Propensity-matched groups (n = 182)		
	MIAVR group (n=91)	CAVR group (n=91)	*P*-value
Mechanical ventilation, hours	6 ± 2	9 ± 2	< 0.01
Reintubation	1 (1.1%)	5 (5.5%)	0.21
CPAP therapy	3 (3.3%)	12 (13.2%)	0.03
Inotropes	16 (17.6%)	14 (15.4%)	0.84
Stroke	1 (1.1%)	1 (1.1%)	0.99
Peripheral vascular complications	1 (1.1%)	-	n.a.
Re-exploration for bleeding	3 (3.3%)	5 (5.5%)	0.72
Sternal complications/mediastinitis	0 (0%)	1 (1.1%)	0.99
Superficial wound complications	2 (2.2%)	5 (5.5%)	0.44
New onset AF	18 (19.8%)	23 (25.3%)	0.48
Need for PMK implantation	4 (4.4%)	2 (2.2%)	0.68
Renal replacement therapy	0 (0%)	0 (0%)	0.99
Red blood cells transfusion	15 (16.5%)	22 (24.2%)	0.27
Hospital stay, days	6.7 ± 2.1	7.2 ± 1.9	0.09
ICU stay, days	1.8 ± 1.2	3.2 ± 1.4	< 0.01
30-day mortality	1 (1.1%)	0 (0%)	0.99

Data were presented as n (%) or mean ± standard deviation; n.a. means not applicable

AF=atrial fibrillation; CAVR=conventional aortic valve replacement; CPAP=continuous positive airway pressure; ICU=intensive care unit; MIAVR=minimally invasive aortic valve replacement; PMK=permanent pacemaker

### Follow-up

**Fig. 1:**
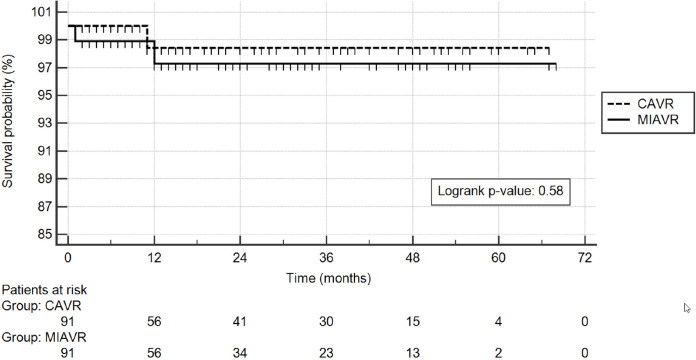
Kaplan Meier follow-up survival curves of the propensity-matched groups. CAVR=conventional aortic valve replacement; MIAVR=minimally invasive aortic valve replacement.

The median follow-up was 17 months (IQR: 8 - 37) and 21 months (IQR: 9 - 40) for the MIAVR and CAVR group, respectively. All patients had postoperative follow-up. As shown by Kaplan-Meier curves (Figure 1), survival rates showed no significant difference between groups (logrank *P*-value = 0.58). No patient was reoperated on during the follow-up. Finally, wound complications requiring surgery occurred in one (1.1%) and two (2.2%) patients in the MIAVR and CAVR groups, respectively (*P*=0.99).

## DISCUSSION

Since it was introduced in the mid-1990s^[15]^, minimally invasive surgery has been associated with many advantages, such as less postoperative bleeding, fewer transfusions, better respiratory recovery, and reduced hospitalisation^[10,16]^. Although these results have not been universally confirmed and a certain degree of uncertainty still exists, the minimally invasive approach has experienced rampant growth and is now widely used^[17]^.

If real, these advantages could be of particular benefit in obese patients, a subgroup at increased risk after cardiac surgery^[5,6]^. On the other hand, some authors have expressed the concern that minimally invasive surgery in the obese could result in unsatisfactory surgical exposure, increased technical complexity of the procedure, and suboptimal outcomes^[9,11]^; moreover, the already long operating time of minimally invasive procedures could further increase with risk of more postoperative complications^[9,11]^.

The aim of our study was to clarify whether a minimally invasive approach to the aortic valve via partial upper V-shaped sternotomy could offer advantages to obese patients compared to the conventional full sternotomy.

In a retrospective study of 613 obese patients, Mikus et al. concluded that minimally invasive aortic valve surgery was associated with reduced mortality, shorter mechanical ventilation times, fewer transfusions, and less need for inotropic support^[18]^. They found no differences in terms of length of hospital stay or wound complications. However, 17% of full sternotomy patients were urgent cases, 21% were redo operations, and 6.2% had active infective endocarditis. In addition, the authors included patients undergoing both partial upper sternotomy and anterior right thoracotomy.

In a previous study, Welp et al. retrospectively compared 217 patients who underwent aortic valve replacement using a minimally invasive approach via partial upper *vs.* full sternotomy^[9]^. They found a shorter duration of mechanical ventilation (6 *vs.* 8 hours) and lower rate of reintubation (0% *vs.* 7%) and tracheostomy (0% *vs.* 4.4%) in the minimally invasive group. Similarly, patients in the mini group had lower transfusion rates and a shorter ICU stay. The conversion rate (0.8%) was low and operating times not significantly prolonged. Also, high-complexity patients were not excluded.

In contrast, Pisano et al. performed a propensity-matched study on 84 patients undergoing aortic valve replacement via J-sternotomy or full sternotomy^[19]^. The authors’ main aim was to understand whether a minimally invasive approach could confer clinical benefit on patients at higher risk, *i.e.*, elderly or severely obese patients. They found that patients above the 4^th^ percentile for BMI had shorter mechanical ventilation times with minimally invasive surgery but comparable postoperative length of stay.

In order to reduce possible confounding factors, we intentionally excluded more complex patients such as redo operations, active endocarditis, and urgent procedures. Furthermore, we only considered patients undergoing isolated aortic valve replacement with a minimally invasive approach via partial upper sternotomy. Finally, we used propensity-matching to make the two groups as homogeneous as possible.

Our main finding was that minimally invasive surgery through partial upper sternotomy was safe and effective in our cohort of obese patients. Then, we observed a shorter duration of mechanical ventilation and a lower rate of postoperative CPAP therapy in the MIAVR group. Although other postoperative complications were comparable, patients in the MIAVR group had a shorter ICU stay. In our opinion, preserving the integrity of both the lower part of the sternum and the xiphoid process is of paramount importance to ensure effective respiratory mechanics and faster recovery of respiratory function. This benefit has been already described in the general population^[13]^ but may become decisive in obese patients. Although not specifically investigated, reduced postoperative pain may also promote the observed favourable respiratory outcome^[20]^. Brown et al. found that patients who underwent a mini-sternotomy aortic valve replacement had a two-hour reduction in ventilation time^[16]^. Similarly, Murtuza et al. reported a mean reduction in ventilation time of 2.86 hours with mini-sternotomy^[10]^. Both studies included obese and non-obese patients. In the present analysis, we reported a three-hour-reduced ventilation time and a substantial reduction of CPAP therapy. Reasonably, the magnitude of these findings does not significantly change the clinical outcome of the patient, but they could support a fast and straightforward patient recovery. Further studies are needed to clarify this issue and definitively understand if a minimally invasive approach would enhance the respiratory outcome of obese patients.

In contrast to our previous studies, we found no differences in terms of postoperative bleeding and need for transfusions, although a trend in favour of minimally invasive surgery was evident^[13]^. As already described, minimally invasive surgery lengthens operating times^[7]^. In the MIAVR group, we found extracorporeal circulation and total surgery times to be 11 and 25 minutes longer, respectively. However, we consider the modest extent of this lengthening to be irrelevant from a clinical point of view. Interestingly, compared to our previous results with minimally invasive surgery, we see longer operating times in obese patients^[13]^. This reflects the undeniably greater technical complexity involved in setting up a minimally invasive procedure in an obese patient. However, the conversion rate was acceptable and always due to the impossibility of percutaneously cannulating the common femoral vein. This step is of utmost importance to clear the surgical field and smoothen the procedure. The presence of the central venous cannula in the setting of a very limited surgical approach makes surgery harder, particularly in the obese in whom anatomic structures are deeper. So, when a percutaneous cannulation is not possible, it is necessary to broaden the skin incision and sternotomy to obtain a satisfactory exposure. However, the groin of the obese patient can be difficult to work in. Surgical exposure of the femoral vessels is not recommended due to the risk of wound complications. On the other hand, percutaneous cannulation is not always easy. As proof of this, in our experience in the general population, we had less frequent difficulties with cannulation of the femoral vein resulting in a lower conversion rate (1.7%) to full sternotomy^[13,14]^. However, in order to reduce complications and increase the success rate, cannulation must be echo-guided. Moreover, when available, radioscopic control of the correct positioning of the venous cannula ensures perfect drainage of the heart and facilitates surgical exposure.

The skin incision must also be slightly modified in the obese patient. Indeed, it is common for the higher position of the diaphragm to push the heart and aortic valve upwards. Therefore, a traditional incision risks being too low and not allowing optimal exposure.

Some authors have chosen a mini-thoracotomy approach in obese patients who are candidates for aortic valve replacement^[8,18]^. Although it is reasonable to expect fewer wound complications and a better respiratory outcome with this technique, it must also be considered that it is not systematically applicable. Indeed, it requires specific anatomical requirements that are not always met in the normal-weight patient. Peripheral cannulation may also be counterproductive. The risk, therefore, is that this technique can only be used in selected cases. However, the most important factor in optimising a procedure and reducing complications is the familiarity one has with it. Rather than resort sporadically to thoracotomy, we preferred to standardise the sternotomy approach and use it systemically. In our experience, this has led to optimal results.

### Limitations

This study is burdened by several limitations. First, it is a single-centre experience on a limited number of patients, so the results cannot be generalised and are influenced by specific local protocols. Second, it is a retrospective study: although propensity-matching made the two comparison groups homogeneous, it cannot replace a randomisation process. Third, the duration of follow-up is rather short, and we limited ourselves to investigating only a few aspects such as survival, need for reintervention, and wound complications. However, some authors hypothesize that obese patients are at a higher risk of patient-prosthesis mismatch due to the large body surface area and the greater difficulty of placing a bigger prosthesis with the minimally invasive approach. Therefore, an echocardiographic follow-up would also have been useful.

## CONCLUSION

Minimally invasive aortic valve surgery via partial upper V-shaped sternotomy in obese patients has been shown to be safe and effective. Although it requires more care, surgical exposure is optimal with this approach. When compared to full sternotomy, it has been shown to require shorter mechanical ventilation and reduced need of postoperative CPAP therapy. The length of stay in the ICU is shortened. These advantages might be particularly important for the obese patient to whom minimally invasive surgery should not be denied. Further dedicated studies are needed to confirm these results.

**Table:** 

Authors’ Roles & Responsibilities	
FC	Substantial contributions to the conception or design of the work; or the acquisition, analysis, or interpretation of data for the work; drafting the work or revising it critically for important intellectual content; agreement to be accountable for all aspects of the work in ensuring that questions related to the accuracy or integrity of any part of the work are appropriately investigated and resolved; final approval of the version to be published
PB	Drafting the work or revising it critically for important intellectual content; final approval of the version to be published
NP	Substantial contributions to the conception or design of the work; or the acquisition, analysis, or interpretation of data for the work; final approval of the version to be published
MN	Substantial contributions to the conception or design of the work; or the acquisition, analysis, or interpretation of data for the work; drafting the work or revising it critically for important intellectual content; final approval of the version to be published
GAC	Drafting the work or revising it critically for important intellectual content; final approval of the version to be published
MG	Substantial contributions to the conception or design of the work; or the acquisition, analysis, or interpretation of data for the work; final approval of the version to be published
SD	Substantial contributions to the conception or design of the work; or the acquisition, analysis, or interpretation of data for the work; final approval of the version to be published
VS	Substantial contributions to the conception or design of the work; or the acquisition, analysis, or interpretation of data for the work; final approval of the version to be published
DD	Substantial contributions to the conception or design of the work; or the acquisition, analysis, or interpretation of data for the work; final approval of the version to be published
MM	Agreement to be accountable for all aspects of the work in ensuring that questions related to the accuracy or integrity of any part of the work are appropriately investigated and resolved; final approval of the version to be published

## References

[1] Poirier P, Giles TD, Bray GA, Hong Y, Stern JS, Pi-Sunyer FX (2006). Obesity and cardiovascular disease: pathophysiology, evaluation,
and effect of weight loss: an update of the 1997 American heart association
scientific statement on obesity and heart disease from the obesity committee
of the council on nutrition, physical activity, and
metabolism. Circulation.

[2] Mariscalco G, Wozniak MJ, Dawson AG, Serraino GF, Porter R, Nath M (2017). Body mass index and mortality among adults undergoing cardiac
surgery: a nationwide study with a systematic review and
meta-analysis. Circulation.

[3] Rahmanian PB, Adams DH, Castillo JG, Chikwe J, Bodian CA, Filsoufi F. (2007). Impact of body mass index on early outcome and late survival in
patients undergoing coronary artery bypass grafting or valve surgery or
both. Am J Cardiol.

[4] Sabatino ME, Yang N, Soliman FK, Chao JC, Ikegami H, Lemaire A (2022). Outcomes of minimally invasive aortic valve replacement in
patients with obese body mass indices. J Card Surg.

[5] Shi N, Liu K, Fan Y, Yang L, Zhang S, Li X (2020). The association between obesity and risk of acute kidney injury
after cardiac surgery. Front Endocrinol (Lausanne).

[6] Liu X, Xie L, Zhu W, Zhou Y. (2020). Association of body mass index and all-cause mortality in
patients after cardiac surgery: a dose-response
meta-analysis. Nutrition.

[7] Shehada SE, Elhmidi Y, Mourad F, Wendt D, El Gabry M, Benedik J (2017). Minimal access versus conventional aortic valve replacement: a
meta-analysis of propensity-matched studies. Interact Cardiovasc Thorac Surg.

[8] Santana O, Reyna J, Grana R, Buendia M, Lamas GA, Lamelas J. (2011). Outcomes of minimally invasive valve surgery versus standard
sternotomy in obese patients undergoing isolated valve
surgery. Ann Thorac Surg.

[9] Welp HA, Herlemann I, Martens S, Deschka H. (2018). Outcomes of aortic valve replacement via partial upper sternotomy
versus conventional aortic valve replacement in obese
patients. Interact Cardiovasc Thorac Surg.

[10] Murtuza B, Pepper JR, Stanbridge RD, Jones C, Rao C, Darzi A (2008). Minimal access aortic valve replacement: is it worth
it?. Ann Thorac Surg.

[11] Tabata M, Umakanthan R, Cohn LH, Bolman RM 3rd, Shekar PS, Chen FY (2008). Early and late outcomes of 1000 minimally invasive aortic valve
operations. Eur J Cardiothorac Surg.

[12] Misfeld M, Borger M, Byrne JG, Chitwood WR, Cohn L, Galloway A (2013). Cross-sectional survey on minimally invasive mitral valve
surgery. Ann Cardiothorac Surg.

[13] Bruno P, Cammertoni F, Rosenhek R, Mazza A, Pavone N, Iafrancesco M (2019). Improved patient recovery with minimally invasive aortic valve
surgery: a propensity-matched study. Innovations (Phila).

[14] Cammertoni F, Bruno P, Pavone N, Farina P, Mazza A, Iafrancesco M (2021). Influence of cardiopulmonary bypass set-up and management on
clinical outcomes after minimally invasive aortic valve
surgery. Perfusion.

[15] Cosgrove DM 3rd, Sabik JF. (1996). Minimally invasive approach for aortic valve
operations. Ann Thorac Surg.

[16] Brown ML, McKellar SH, Sundt TM, Schaff HV. (2009). Ministernotomy versus conventional sternotomy for aortic valve
replacement: a systematic review and meta-analysis. J Thorac Cardiovasc Surg.

[17] Nguyen TC, Terwelp MD, Thourani VH, Zhao Y, Ganim N, Hoffmann C (2017). Clinical trends in surgical, minimally invasive and transcatheter
aortic valve replacement†. Eur J Cardiothorac Surg.

[18] Mikus E, Calvi S, Brega C, Zucchetta F, Tripodi A, Pin M (2021). Minimally invasive aortic valve surgery in obese patients: can
the bigger afford the smaller?. J Card Surg.

[19] Pisano C, Totaro P, Triolo OF, Argano V. (2017). Advantages of minimal access versus conventional aortic valve
replacement in elderly or severely obese patients. Innovations (Phila).

[20] Phan K, Xie A, Di Eusanio M, Yan TD. (2014). A meta-analysis of minimally invasive versus conventional
sternotomy for aortic valve replacement. Ann Thorac Surg.

